# *Astragalus mongholicus* Bunge and Panax notoginseng formula (A&P) improves renal mesangial cell damage in diabetic nephropathy by inhibiting the inflammatory response of infiltrated macrophages

**DOI:** 10.1186/s12906-021-03477-x

**Published:** 2022-01-20

**Authors:** Xiao Lin, Xiao-qin Lei, Jie-ke Yang, Jian Jia, Xia Zhong, Rui-zhi Tan, Li Wang

**Affiliations:** Research Center of Intergated Traditional Chinese and Western Medicine, Affiliated Traditional Medicine Hospital, Southwest Medical University, 319# zhongshan road, Luzhou, 646000 Sichuan China

**Keywords:** Diabetic nephropathy, Inflammation, Macrophages, Astragalusmongholicus Bunge and Panax notoginseng formula, Mincle, NF-κB

## Abstract

**Background:**

Diabetic nephropathy (DN) is one of the main causes of end-stage renal disease with scantly effective treatment. Numerous evidences indicated that macrophages play an important role in the occurrence and pathogenesis of DN by secreting inflammatory cytokines. Mincle is mainly expressed in macrophages and promotes kidney inflammation and damage of acute kidney injury. However, the role of Mincle in DN is unclear. In this study, we aim to investigate the effect of Mincle-related macrophage inflammation on DN, and whether it can be identified as the therapeutic target for *Astragalus mongholicus* Bunge and Panax notoginseng Formula (A&P), a widely used Chinese herbal decoction for DN treatment.

**Methods:**

In vivo experiments high-fat and high-sugar diet and streptozotocin was used to establish a diabetic nephropathy model, while in vitro experiments inflammation model was induced by high-glucose in mouse Bone Marrow-Derived Macrophages (BMDM) cells and mouse mesangial (MES) cells. Kidney pathological staining is used to detect kidney tissue damage and inflammation, Western blotting, Real-time PCR and ELISA are performed to detect Mincle signaling pathway related proteins and inflammatory cytokines.

**Results:**

Mincle was mainly expressed in infiltrated macrophage of DN kidney, and was significant decreased after A&P administration. The in vitro experiments also proved that A&P effectively down-regulated the expression of Mincle in macrophage stimulated by high glucose. Meanwhile, the data demonstrated that A&P can reduce the activation of NFκB, and the expression and secretion of inflammatory cytokines in DN kidney or BMDM cells. Notably, we set up a co-culture system to conform that BMDM cells can aggravate the inflammatory response of mesangial (MES) cells under high glucose stimulation. Furthermore, we found that the anti-injury role of A&P in MES cells was dependent on inhibition of the Mincle in macrophage.

**Conclusion:**

In summary, our study found that A&P is effective in reducing renal pathological damage and improving renal function and inflammation in diabetic nephropathy by a mechanism mainly related to the inhibition of the Mincle/Card9/NFκB signaling pathway.

**Supplementary Information:**

The online version contains supplementary material available at 10.1186/s12906-021-03477-x.

## Background

Diabetic nephropathy (DN) is a highly prevalent complication of diabetes and major cause of end-stage renal disease that seriously affects human health in many developed and developing regions [1, 2]. In 2015, there were an estimated 415 million people with diabetes worldwide, 5 million of whom died from diabetes-related complications, and by 2040 there will be 642 million people with diabetes, 30–40% of whom will develop to diabetic nephropathy [3]. The pathological features of early diabetic nephropathy mainly include tubular damage, glomerular hypertrophy, glomerular basement membrane thickening, and loss of podocytes. In the late stage, it mainly includes glomerulosclerosis and renal interstitial fibrosis [4, 5]. Although hyperglycemia, oxidative stress and renin–angiotensin–aldosterone system (RAAS) activation promote renal damage in diabetic nephropathy, substantial evidence suggests that inflammation is a major trigger for the development and progression of diabetic nephropathy [5, 6]. Numerous studies in recent years have shown that the infiltration of multiple immune cells in the kidney of patients with diabetic nephropathy is significantly increased, exacerbating the inflammation of kidney. Notably, macrophages are the most infiltrated immune cells in the kidney of patients with diabetic nephropathy, and mainly infiltrated in glomeruli and interstitium [7–9]. In the past decades, the only treatment strategy considered effective for diabetic nephropathy was blockade of the renin–angiotensin system (RAS), but many patients still develop to chronic kidney disease (CKD) or end-stage renal disease (ESRD). Therefore, anti-inflammatory treatment may be a new direction for future DN treatment [10].

In the early stage of diabetic nephropathy, macrophages are derived from recruited monocytes, which produce a variety of molecules to promote renal injury, including pro-inflammatory cytokines, reactive oxygen species, complement systemic factors, and chemokines [11, 12]. Macrophage infiltration and activation are significantly associated with glomerular damage and endothelial activation, and induce proteinuria and glomerulosclerosis [13, 14]. Notably, previous studies reported that macrophages promote mesangial fibrosis and proliferation in DN [15]. Macrophage polarization plays an important role in the early stage of DN kidney, among which the proinflammatory M1 macrophages increase rather than the anti-inflammatory M2 macrophages, which accelerates the damage of the DN kidney [16]. In DN, M1 macrophages release TNF-α, which promotes renal inflammation through NF-κB, JAK and other signaling pathways, and directly interferes with podocyte integrity [17, 18]. Mincle is a pattern recognition receptor expressed mainly on membrane of macrophage and plays a key role in inflammatory response of macrophage [19, 20]. A recent study reported that Mincle maintains the phenotype of M1 macrophage in acute kidney injuery (AKI) [19] and inhibition of Mincle as well as its signaling pathway in macrophage ameliorates the renal injury in AKI [21, 22], suggeting that Mincle is a potential target for the treatment of AKI. However, the role of Mincle in DN is unclear. Suppression of Mincle and Mincle-maintained M1 macrophage polarization may be a potential target for the treatment of DN, given the pro-inflammatory role of M1 macrophages in DN.

*Astragalus mongholicus* Bunge and Panax notoginseng formula (A&P), composed of Panax notoginseng, *Astragalusmongholicus* Bunge, Angelica sinensis, *Achyranthes bidentata*blume, and *Ecklonia kurome* Okamura, is a traditional Chinese herbal medicine widely used in Southwest of China for the treatment of chronic kidney disease [23]. Our previous studies demonstrated that A&P protects kidney from inhibiting inflammation through downregulating of Mincle pathway in macrophage in AKI [24]. Moreover, recent study has showed that A&P combined with Bifidobacterium can protect kidney from CKD by inhibiting Mincle, that downregulated the inflammatory response of macrophage in kidney and intestine [25]. Since A&P can inhibit the expression of Mincle in AKI and CKD kidneys, whether it has a protective effect on DN kidneys, and whether it can protect glomerular mesangial cells from hyperglycemia by inhibiting Mincle maintained inflammatory response in macrophage, which will be the main purpose of this research.

This study examined the effects of A&P on renal injury in DN, and studied the effects of macrophagic Mincle on the inflammation and injury of mesangial cells through cellular co-culture technology. The protective effect of A&P on mesangial cells was investigated by down-regulating and overexpressing Mincle in macrophages through gene modification techniques. This study may provide a potential therapeutic target for DN.

## Methods

### *Astragalus mongholicus* Bunge and Panax notoginseng formula (A&P)

*Astragalus mongholicus* Bunge and Panax notoginseng Formula (A&P) used in this study is not a mixture of Chinese herbal decoction but a boil-free granule of traditional Chinese medicine. The mian regent of A&P consist of 3 g of Angelica sinensis, 3 g of *Achyranthes bidentata*, 3 g of Ecklonia kurome, 3 g of Astragalus propinquus Schischkin and 1 g of Panax notoginseng. The procurement and production of drugs are completed by the Chinese Pharmacy of Affiliated Traditional Chinese Medicine Hospital of Southwest Medical University. The dose of A&P used in the in vivo experiments is determined by the transformation formula between humans and experimental animals. In clinical treatment, the daily dose of A&P for adults is 13 g/day. According to the human-to-mouse conversion ratio (9.1, 23], the daily A&P dose (g/kg) of mice can be calculated by the fllowing: Daily dose of mice = conversion ratio between mice and humans (9.1) × a daily dose of an adult (13 g)/average weight of adults (60 kg) = 9.1 × 13 (g)/60 (kg) = 1972 mg/kg. The minimum daily dose of mice was set as the low-dose intervention group, and the high-dose was four times of the low-dose (7888 mg/kg).

### Preparation of A&P

Mice in experimental groups were fed with hand-made jelly containing A&P. Briefly, purified water was boiled and mixed with edible gelatin, normal mouse feed powder and A&P, followed by cooling in a 24-well plate. In vitro experiments, serum containing A&P was used to protect cells from the interference of Chinese herbal medicine impurities. Male C57BL/6 mice were administered with A&P continuously for 7 days. On the 7th day, the mice were sacrificed and the blood was collected. The blood samples were placed in a refrigerator at 4 °C overnight and then centrifuged at 3000 rpm/min for 20 min at 4 °C. Collect serum for subsequent cell experiments.

### Extraction of mouse bone marrow-derived macrophages (BMDM)

After sacrificing the male C57BL/6 mice, the bodies were immersed in 70% alcohol for 5 min for sterilization. Subsequently, the hip joint, pubic bone, femur, tibia, and proximal foot bone were collected, then the attached muscles were removed by scissors carefully. The bones were placed in a petri dish containing sterilized phosphate buffered saline (PBS), followed by sterilizing in 70% alcohol for 30s, and then washing with PBS again. The sterilized bones were transferred to a mortar with sterilized PBS and then grinded by a lapping rod. The liquid was transfered to 50 mL centrifugal tube with a 70 μm filter, then centrifuged at 1500 rpm for 5 min, and the supernatant was removed. Add 9 mL RO water and 1 mL 10 × PBS to remove the red blood cells. After filtering with a 40 μm filter, centrifuge at 1500 rpm for 5 min, followed by resuspend cells and cultured in DMEM complete medium containing 30% L-929 cell supernatant for 7 days to differentiate macrophages.

### Cell culture

The mouse mesangium cells (MES) were cultured in Roswell Park Memorial Institute (RPMI) 1640 medium containing 10% FBS (Gibco, 10,099-141C, USA), 100 U/ml penicillin and 100 μg/ml streptomycin at 37 °C with 5% CO_2_. The BMDM cells were cultured in Dulbecco Modified EagleMedium (DMEM, Sigma-Aldrich, St. Louis, MO, USA) supplementedwith 30% L929 supernatant, 10% FBS, 100 U/ml penicillin and 100 μg/ml streptomycin at 37 °C with 5% CO_2_.

### Co-culture system

BMDM and MES cells at the logarithmic growth stage were co-cultureed by using Transwell chamber and 6-well cultured plate (Millicell, PIHT30R48, USA). The BMDM cells were inoculated into the Transwell chamber at 2 × 10^5^ /well as the upper of the co-culture system, and the MES cells were inoculated at 5 × 10^5^ /well in the 6-well culture plate. The co-cultured cells were incubated for 12 h in RPMI 1640 medium without FBS, and 35 mM of D-(+)-glucose (Sigma, G7021-100G, USA) was added to construct the inflammatory cell model.

### Animals experiments

Male C57BL/6 mice (6–8 weeks) with weight of 19 ± 1 g were purchased from Chengdu Dashuo Experimental Animal Co. Ltd., and raised in the center of laboratory animals of Southwest Medical University with room temperature 20–23 °C, relative humidity 50–60% and 12 h alternating light and dark. Ten mice were used as normal control group (NC), forty mice were used for modeling. After all the mice were adaptively fed for 2 weeks, the mice in the normal group were still fed standard chow as before, while the mice in the diabetic nephropathy group and intervention groups were fed high-fat and high-sugar diet for 8 weeks. Then, after 12 h of fasting without water, a single intraperitoneal injection of 50 mg/kg of Streptozotocin (Sigma, S0130, USA) was injected into the special diet groups for 5 consecutive days in order to destroy the β-cells. The normal group was injected with an equal volume of citrate buffer at the same time. One week later, the fasting blood glucose level was measured through tail vein blood. It was considered as a successful model of diabetic mice when the glucose value of fasting blood was over 11.1 mmol/L. After 3 weeks, the real-time detection of 24 h proteinuria was performed on these models, in which it was considered as DN model when the 24 h proteinuria was more than 30 mg. Subsequently, these models were randomly divided into 4 groups: diabetic nephropathy (DN), low A&P interventiongroup (A&P L, 1972 mg/kg), high A&P intervention group (A&P H, 7888 mg/kg), and positive control group (IRB, 20 mg/kg). All mice in the diabetic nephropathy group and the treatment groups continued to be given high-fat and high-sugar diet during the 8-week A&P intervention and were weighed once every 2 weeks. After the experiment, the blood and kidney of the mice were collected, and the tissues were fixed in 10% neutral formalin or stored at − 80 °C for later use.

### Determination of cell viability

3-(4,5-Dimethylthiazole-2-yl)-2,5-diphenyltetrazolium bromide (MTT) (Solarbio, M8180, China) was used to determine the effect of A&P-containing serum on cell viability. After starving overnight in 1640 RPMI medium without FBS, the primary mouse macrophages and mouse mesangial cells (2 × 10^3^ cells/well) grown in 96-well plates were treated with A&P-containing serum (5, 10, 20, 25, 30 and 35%) for 24 h. 10 μL of 5 mg/mL MTT solution was added to each well and further cultured in an incubator containing 5% CO2 at 37 °C for 4 h. Subsequently, 100 μL of dimethyl sulfoxide was added to each well, and the OD value was detected with a microplate reader at 570 nm. The cell Viability was calculated as a percentage according to the instructions provided by the manufacturer.

### Detection of renal function and physiological function

After taking blood from the heart of the mice in each group, the collected blood was kept at 4 °C overnight, and the next day, the serum on the top was obtained by centrifuging at 3000 r/min for 15 min. The serum samples were performed urea nitrogen (BUN) (Nanjing JianCheng, C013–2-1, China) and creatinine (CREA) (Nanjing JianCheng, C011–2-1, China) test with kits. 24 h urine samples from mice in each group was collected to detect the urine protein (Nanjing JianCheng, C035–2-1, China). After fasting for 6 h, fasting blood glucose was tested by blood glucose test strips (Omron, Japan).

### H&E Staining and PAS staining

The fresh kidney tissue was fixed with 10% neutral formalin solution and embedded in paraffin before cuting into 4 μm slices. After baking slices at 65 °C for 1.5 h, the slices are dewaxed in xylene and rehydrated in gradient alcohol, and then stained with H&E (Beyotime, C015, China) and PAS (Solarbio, G1285, China) solution in strict accordance with the manufacturer’s instructions.

### Immunohistochemical staining

For immunohistochemistry, TNF-α, IL-1β, IL-6, and F4/80 were tested in kidney samples on slices. First, the slices were dewaxed in xylene and rehydrated in gradient alcohol before antigen repair (citrate buffer pH 6.0, 95 °C for 10 min). Next, immunohistochemical staining of the tissue was performed according to the kit instructions. Briefly, the slices were incubated at 4 °C overnight with corresponding primary antibody (TNF-α, Santa, sc-52,746, USA, 1:200; IL-1β, Santa, sc-12,742, USA, 1:200; IL-6, Santa, sc-532,296, USA, 1:200; F4/80, Santa, sc-377,009, USA, 1:200) in 1% bovine serum albumin (BSA). The next day, after washing three times with PBS, the slices were treated with Biotin-Streptavidin HRP-based SPlink Detection Kits (ZSGB-Bio, PV-6000, China) and stained the nucleus with hematoxylin. Ultimately, all the slices were photographed with Virtual Slide Microscope (VS120-S6-W, Olympus, Japan).

### Immunofluorescence staining

For immunofluorescence, the kidney tissues were fixed in 10% formaldehyde solution at 4 °C for 24 h, and were respectively dehydrated with 10, 20, 30% sucrose, followed by embedding in optimal cutting temperature compound (OCT). Then the 4 μm slices of kidney samples were incubated with primary antibody (F4/80, Santa, sc-377,009, USA, 1:200; Mincle, Santa, sc-390,806, USA, 1:200) at 4 °C overnight. After washing 3 times with PBS for 5 min, samples were incubated with Alexa Fluor® 488 (Cell Signaling Technology, #8878, USA; 1:200) and Alexa Fluor® 647 (Cell Signaling Technology, #8940, USA; 1:200) conjugated secondary antibodies at room temperature for one hour the next day. Finally, take the images with Virtual Slide Microscope (VS120-S6-W, Olympus, Japan).

### ELISA assay

The concentration of TNF-α, IL-1β, IL-6 in the supernatant of each group were detected by enzyme linked immunosorbent assay (ELISA) kit (Elascience, E-EL-M0044c, E-EL-M0037c, E-EL-M0049c, China). The supernatant of each group was collected to detect the concentration of inflammatory cytokine according with the ELISA kit instructions. Subsequently, the OD value was measured at 450 nm and the actual concentration of each cytokine was calculated by the standard curve.

### RNA isolation and quantitative PCR

Total RNA from BMDM, MES and mouse kidney tissues were isolated by using TRIzol Reagent (TianGen, DP419, China). The reverse transcription was performed to obtain complementary DNA (cDNA) by using the Reverse Transcription Kit (Promega, A1260, China). Finlly, quantitative PCR was performed using Eastep qPCR Master Mix (Promega, A1260, China) on the LightCycler® 480 II Real-Time PCR System (Roche, Germany). The primers were synthesized by Sangon Biotech (Shanghai, China), and the sequences are shown in Table 1.

**Table 1:** The primer sequences for Real-Time PCR

Gene	GenePrimer sequence (5′-3′)
F4/80	F:TGACTCACCTTGTGGTCCTAA
	R:CTTCCCAGAATCCAGTCTTTCC
TNF-α	F:CATCTTCTCAAAATTCGAGTGACAA
	R:TGGGAGTAGACAAGGTACAACCC
IL-1β	F:TGCCACCTTTTGACAGTGATG
	R:AAGGTCCACGGGAAAGACAC
IL-6	F:AAAGAGTTGTGCAATGGCAATTCT
	R:AAGTGCATCATCGTTGTTCATACA
Mincle	F:ACCAAATCGCCTGCATCC
	R:CACTTGGGAGTTTTTGAAGCATC
β-actin	F:GGCTGTATTCCCCTCCATCG
	R:CCAGTTGGTAACAATGCCATGT

### Western blotting

Total protein form cells and kiney tissues were isolated with RIPA buffer, and protein concentrations were measured by using a BCA Protein Assay Kit (Beyotime, P0012, China). Protein samples were separated on 12% SDS-Polyacrylamide Gel and transferred to polyvinylidene fluoride (PVDF) membranes (Millipore, IPVH00010, USA). After blocking with 5% bovine serum albumin at room temperature for 1 h, the PVDF membranes were incubated at 4 °C overnight with the primary antibodies anti-β-actin (Invitrogen, 14–9760-82, USA, 1:10000), anti-IL-1β (Santa, sc-12,742, USA, 1:1000), anti-TNF-α (Santa, sc-52,746, USA, 1:1000), anti-IL-6 (Santa, sc-532,296, USA, 1:1000), anti-Card9 (Santa, sc-374,569, USA, 1:1000), anti-NFκB (Santa, sc8008, USA, 1:1000), anti-p-NFκB (Santa, sc166748, USA, 1:1000), anti-Mincle (Santa, sc-390,806, USA, 1:1000). After washing with TBST for 5 min thrice, PVDF membranes were incubated with secondary antibody (Invitrogen, A32723, USA, 1:10000) at room temperature for 1 h. Bands were visualized using ChemiDoc™ (Bio-Rad, USA) and gray intensity of the bands were analyzed using ImageJ software.

### Plasmid and siRNA transfection

The *Escherichia coli* containing Mincle overexpression plasmid pcDNA3.1-Mincle was purchased from GenePharma company, and the plasmid was extracted from *Escherichia coli* by using a plasmid extraction kit (NucleoBond Xtra MiDi EF, 740420.50, Germany). siRNA-Mincle was synthesized by Sangon Biotech (Shanghai, China) and sequences are available in Table 2. The plasmid or siRNA and the ZETA transfection reagent were mixed for 10 min, next, DNA-lipid complex was added to the medium of each group for 24 h. The transfection efficiency can be observed using a fluorescence microscope.

**Table 2:** The sequences of siRNA-Mincle

Name	Sequence (5′-3′)
siRNA-Mincle	S:CCUUUGAACUGGAAACAUUTT
	A:AAUGUUUCCAGUUCAAAGGTT

### Statistical analysis

All the data of this study were shown as mean ± SD. Statistical analyses were performed with one-way analysis of variance (ANOVA) using a SPSS 21.0 Software (SPSS, Inc., Chicago, IL, USA). *P*<0.05 was means to be a statistical difference.

## Results

### A&P prevented the progression of diabetic nephropathy

**Fig. 1:**
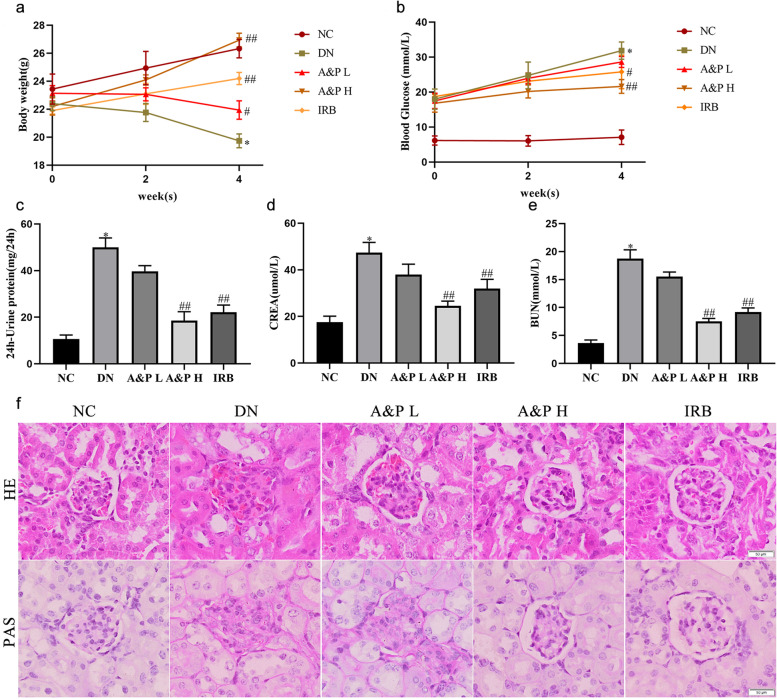
A&P prevented the progression of diabetic nephropathy. **a-b**, the effect of A&P on body weight and fasting blood glucose in DN mice. **c-e**, A&P admnistration effectivle reduced the levels of serum creatinine (CREA), serum urea nitrogen (BUN), and 24 h-Urine protein in DN mice. **f**, HE staining, PAS staining of mouse kidney (× 200). (The data are expressed as the mean ± SD. Compared with NC group, *P < 0.01. Compared with DN group, ^#^*P* < 0.05, ^##^*P* < 0.01)

To investigate the effect of A&P on DN, the body weight, 24 h-urine protein, serum creatinine and serum BUN were measured. The results showed that treatment with A&P strongly increased the body weight in diabetic nephropathy mice (Fig. 1A). Surprisingly, fasting blood glucose, the most important indicator of diabetic nephropathy, was lowered after A&P intervention (Fig. 1B). Notably, the results of renal function detection demonstrsated that the administration of A&P can also obviously reduce the levels of serum creatinine (CREA), serum urea nitrogen (BUN), and 24 h-urine protein compared with DN mice, it clearly reflected that A&P improved renal fuction in DN mice (Fig. 1C-E). Histological examination of kidney from DN mice by H&E and PAS staining showed mesangial expansion, thickened basement membrane, focal glomerulosclerosis, as well as increased glomerular matrix, glycogen vacuoles and mucus. After treatment by A&P, the kidney injury has been significantly improved (Fig. 1F). These findings confirmed that A&P prevented the progression of diabetic nephropathy.

### A&P prevented renal inflammation in diabetic nephropathy mice

**Fig. 2:**
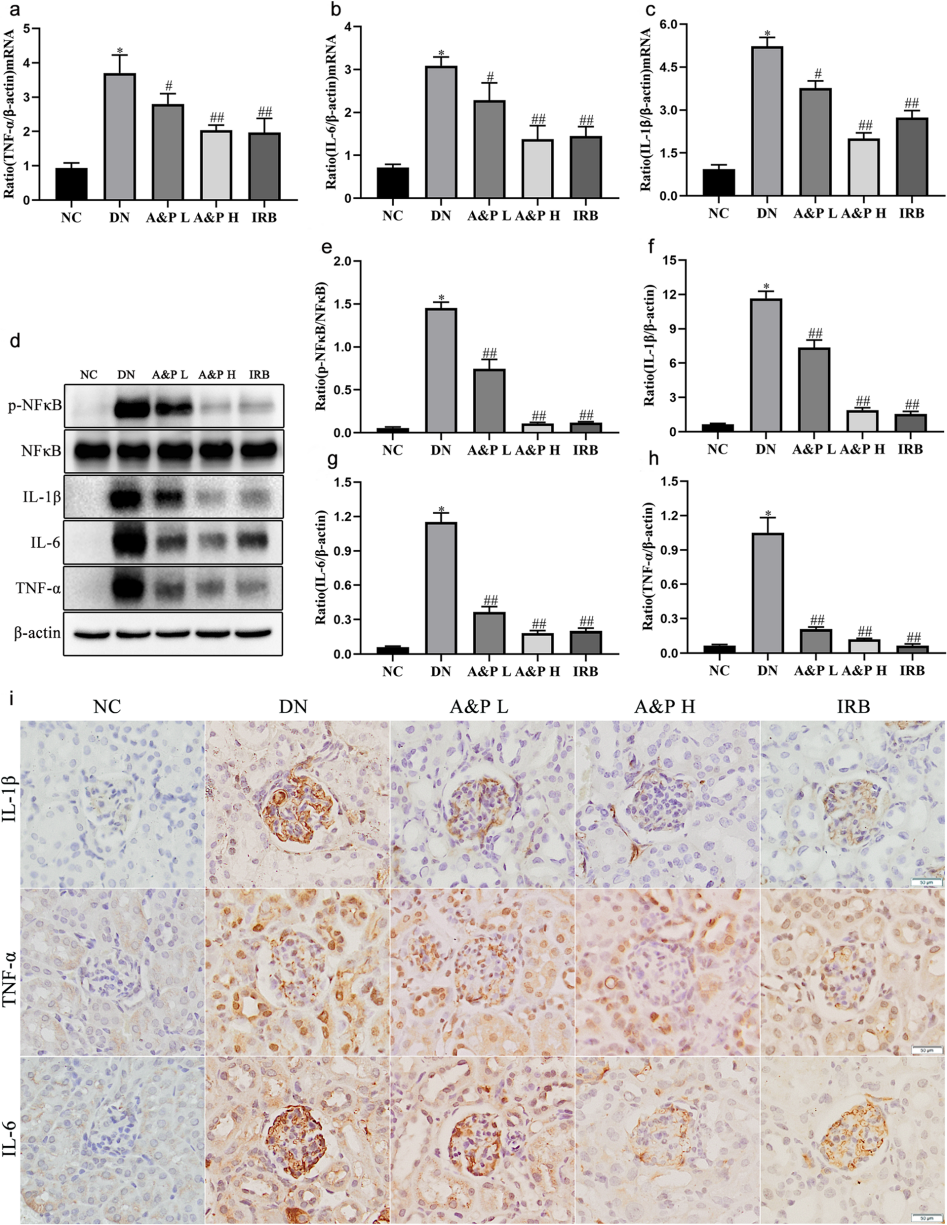
A&P prevented renal inflammation in diabetic nephropathy mice. **a-c**, A&P admnistration effectivly reduced the mRNA expression of TNF-α, IL-1β and IL-6 in DN mice. **d-h**, The relative expression of p-NFκB detected by western boltting analysis normalized to NFκB expression, and the relative expression of TNF-α, IL-1β and IL-6 detected by western boltting analysis normalized to β-actin expression. **i**, Immunoflfluorescence staining of TNF-α, IL-1β and IL-6 in kidney of DN mice. (The data are expressed as the mean ± SD. Compared with NC group, *P < 0.01. Compared with DN group, ^#^*P* < 0.05, ^##^*P* < 0.01)

Inflammatory reactions are a consistent pathological characteristic of diabetic nephropathy, in which the activation of NF-κB leads to the release of inflammatory factors. Therefore, we detected the mRNA and protein levels of inflammatory cytokines and NF-κB using Real-time PCR, western botting and immunohistochemistry. The results showed that the mRNA expression of TNF-α, IL-1β and IL-6 was elevated in DN mice compared with the normal mice, while treatment with A&P remarkably reduced the expression levels of these cytokines (Fig. 2A-C). Similar results were revealed by western botting that A&P can inhibit the activation of NFκB and down-regulate the protein levels of TNF-α, IL-1β and IL-6 in kidney of DN mice (Fig. 2D-H). In addtion, IHC staining also revealed that TNF-α, IL-1β and IL-6 signifificantly increased in diabetic nephropathy kidney, its secretion can be suppressed by treatment of A&P (Fig. 2I). All the above results implys an anti-inflammatory effect of A&P in diabetic nephropathy.

### A&P reduced the expression of Mincle in macrophages of diabetic nephropathy mice

**Fig. 3:**
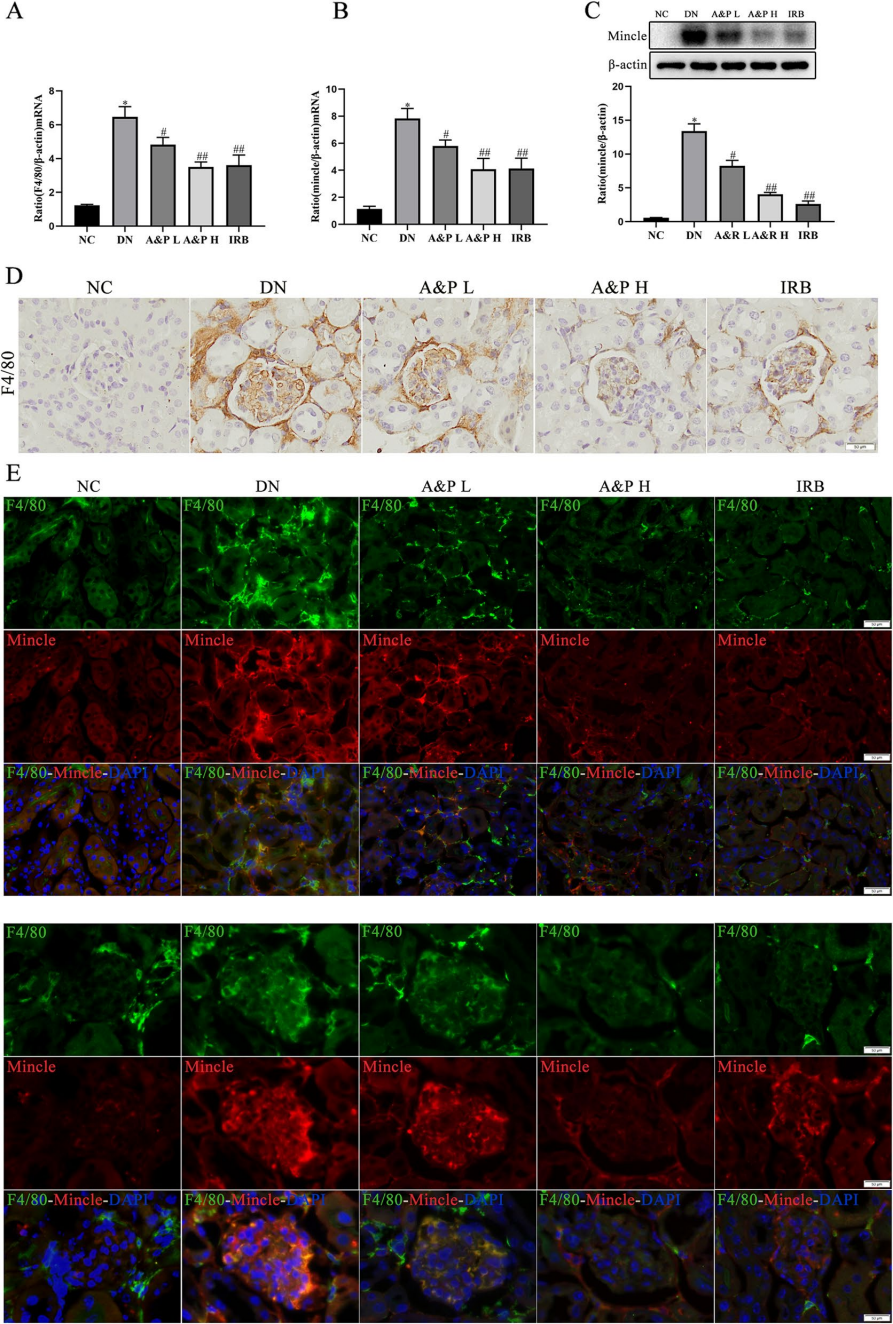
A&P reduced the expression of Mincle in macrophages of diabetic nephropathy mice. **a-b**, Effect of A&P on the mRNA expression of F4/80 and Mincle in DN mice detected by Real-time PCR analysis. **c**, The relative expression of Mincle detected by western boltting analysis normalized to β-actin expression. **d**, Immunoflfluorescence staining of F4/80 in kidney of DN mice. (The data are expressed as the mean ± SD. Compared with NC group, *P < 0.01. Compared with DN group, ^#^*P* < 0.05, ^##^*P* < 0.01)

Mincle is a germline-encoded transmembrane pattern recognition receptor in kidney inflammation, and it combines with damage-related molecular pattern proteins and pathogen-related molecular pattern proteins to exert immune effects. Previous reports indicated that down-regulation of Mincle in renal macrophages can reduce the renal inflammation of AKI, however, the effect of Mincle on kidney of DN is unknown. In this study, we then examed the macrophage infiltration in kidney of DN by detection the expression of F4/80, a typical macrophage marker. The expression of F4/80 was significantly increased in kidney of DN, which was consistent with the results of F4/80 immunohistochemistry, suggesting that macrophages were largely infiltrated in kidney of DN (Fig. 3A, D). Notably, treatment with A&P prevented the infiltration of macrophage in kidney of DN. On the other hand, the mRNA and protein levels of Mincle were largely increased in kidney of DN mice, which were down-regulated in kidney after A&P treatment (Fig. 3B-C). The immunofluorescence was performed to co-locate the Mincle and macrophage, the results revealed that macrophages (green spots) infiltrated in DN kidney, while Mincle (red spots) was co-located with F4/80, suggesting that Mincle expresses on macrophage (Fig. 3E). Interestingly, the results of IF showed that Mincle is also highly expressed in glomerulus, suggesting that Mincle may induce the damage of glomerular mesangial cells in DN. All the results clearly indicated that Mincle is mainly expressed on the macrophage of kidney in DN mice, moreover, A&P remarkably reduced the expression of Mincle in macrophages of diabetic nephropathy mice.

### A&P inhibits Inflammaion in BMDM cells

**Fig. 4:**
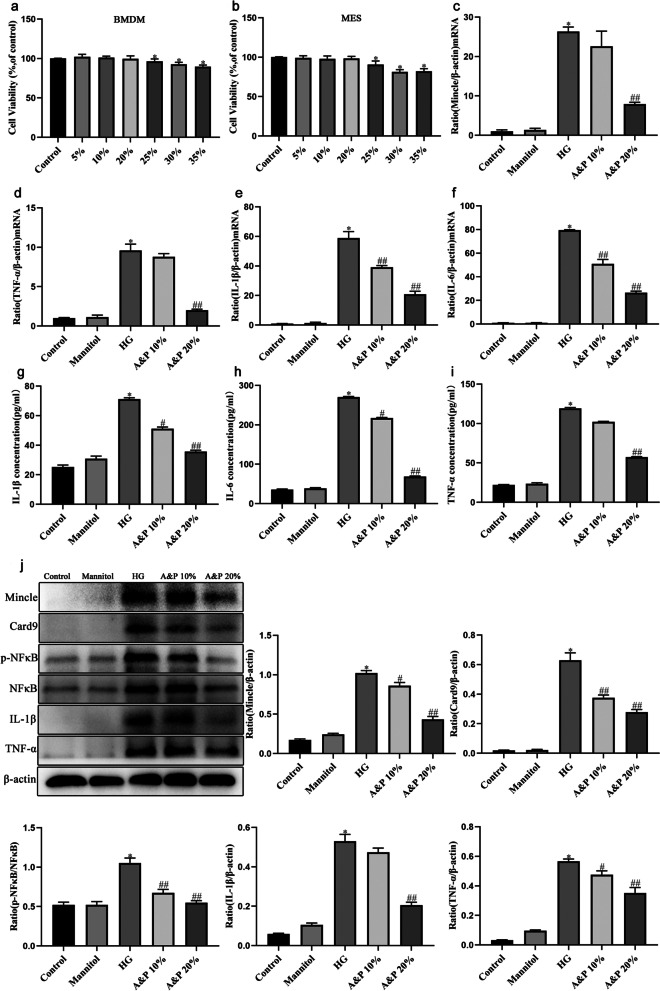
A&P inhibits inflammaion in BMDM cells. **a-b**, MTT viability assay. **c-f**, Effect of A&P on the mRNA expression of Mincle, TNF-α, IL-1β and IL-6 in HG-induced BMDM cells according to Real-time PCR analysis. **g-i**, the content of TNF-α, IL-1β and IL-6 in supernatant in HG-induced BMDM cells detected by ELISA assay. **j**, The relative expression of p-NFκB detected by western boltting analysis normalized to NFκB expression, and the relative expression of Mincle, Card9, TNF-α, IL-1β and IL-6 detected by western boltting analysis normalized to β-actin expression. (The data are expressed as the mean ± SD. Compared with Control group, *P < 0.05. Compared with HG group, ^#^*P* < 0.05, ^##^*P* < 0.01)

We next examined the effects of A&P on inhibiton of Mincle/Card9/NF-κB signaling pathway in BMDM cells. Firstly, the cytotoxicity of A&P-containg serum on BMDM cells and MES cells were detected by using MTT assay and we determined the final concentration of A&P-containg serum was 10 and 20% (Fig. 4A, B). The real-time PCR and ELISA results showed that the expression and secretion of inflammatory cytokines were significantly reduced after A&P administration (Fig. 4C-I). Moreover, western blotting results demonstrated that A&P obviously down-regulated the protein level of Mincle in HG-reduced BMDM, and also down-regulated the downstream indicators of Mincle, such as Card9 and p-NFκB, as well as the inflammatory cytokines, TNF-α, IL-1β and IL-6 (Fig. 4J). These findings revealed that A&P inhibits Mincle/Card9/NF-κB pathway and inflamation in BMDM cells.

### HG-induced BMDM aggravated the inflammatory response of MES cells

**Fig. 5:**
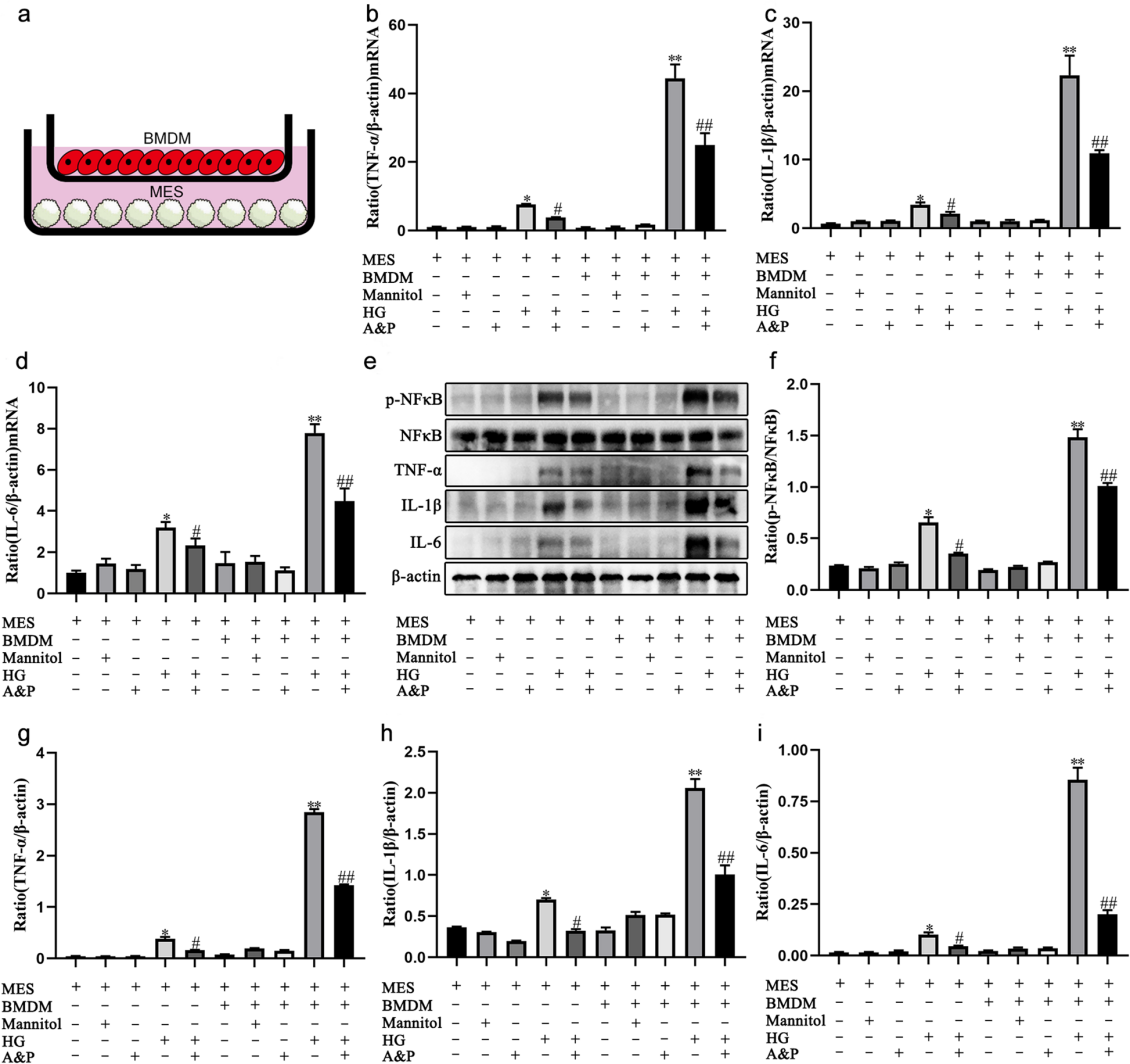
HG-induced BMDM aggravated the inflammatory response of MES cells. **a**, The schematic diagram of the co-culture system. **b-d**, Effect of A&P on the mRNA expression of TNF-α, IL-1β and IL-6 in HG-induced MES cells and co-culture cells detected by Real-time PCR analysis. **e-i**, Effect of A&P on the protein level of p-NFκB, TNF-α, IL-1β and IL-6 in HG-induced MES cells and co-culture cells detected by western blotting analysis. (The data are expressed as the mean ± SD. Compared with Control group of single culture, *P < 0.01. Compared with HG group of single culture, ^#^*P* < 0.01. Compared with HG group of single culture, **P < 0.01. Compared with HG group of co-culture, ^#^*P* < 0.01)

To explore the effect of HG-induced BMDM on MES cells injury, we set up a co-culture system. As shown in Fig. 5A, the BMDM cells were cultured in an upper transwell chamber while the MES cells were cultured in a well of plate. The results of real-time PCR demonstrated that the mRNA levels of TNF-α, IL-1β and IL-6 in mesangial cells co-cultured with HG-induced BMDM were significantly elevated than those groups without BMDM (Fig. 5B-D). Notably, A&P can reduce the expression of inflammatory cytokines in mesangial cells co-cultured with or without macrophages. At the same time, we also examined the protein levels of p-NFκB, TNF-α, IL-1β and IL-6 by western blotting, and the results were the same as the real-time PCR (Fig. 5E-I). In brief, BMDM cells can aggravated the inflammatory response of mesangial cells under high glucose stimulation.

### Mincle is the key to A&P to improve mesangial cell damage by Down-regulating macrophage inflammation

**Fig. 6:**
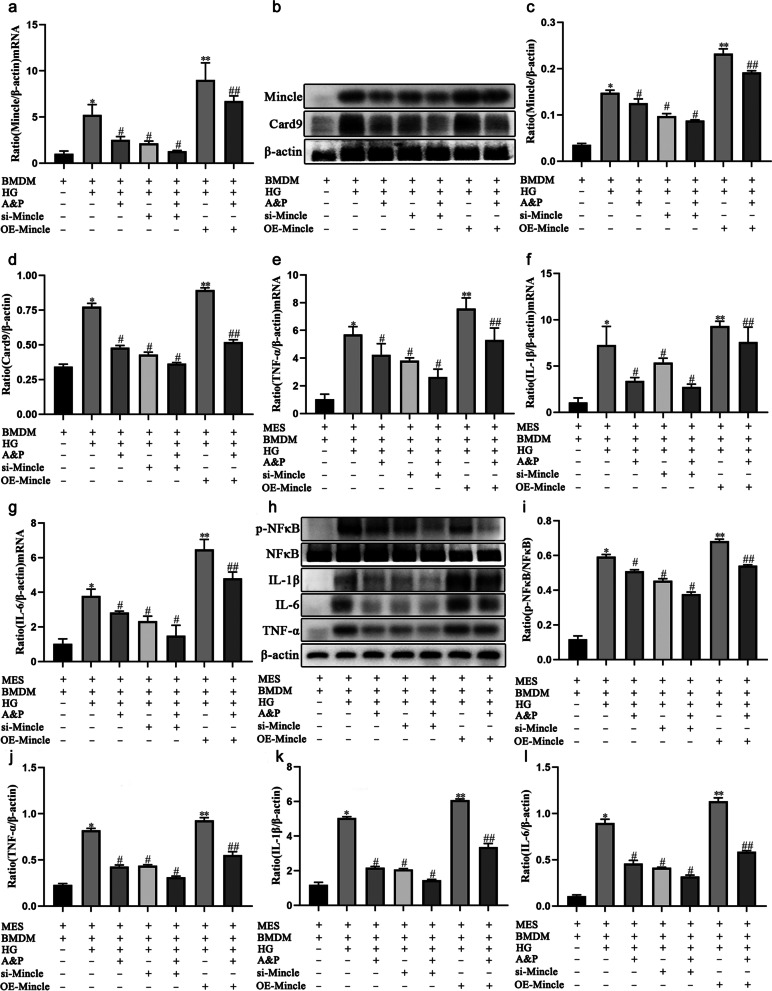
Mincle is the key to A&P to improve mesangial cell damage by down-regulating macrophage inflammation. **a**, The mRNA expression of Mincle in BMDM cells detected by Real-time PCR analysis. **b-d**, The protein level of Mincle in BMDM cells detected by western boltting analysis. **e-g**, The mRNA expression of TNF-α, IL-1β and IL-6 in co-culture cells detected by Real-time PCR analysis. **h-l**, The protein level of p-NFκB, TNF-α, IL-1β and IL-6 in co-culture cells detected by western boltting analysis. (The data are expressed as the mean ± SD. Compared with Control group, *P < 0.01. Compared with HG group, ^#^*P* < 0.05, **P < 0.05. Compared with HG + A&P group, ^##^*P* < 0.05)

To futher elucidate the therapeutic mechanism of A&P in DN, we knockdown and overexpress Mincle in A&P treated HG-induced BMDM cells by siRNA and plasmid transfection, and co-cultured with MES cells (Supplementary Fig. 1). Real-time PCR and western blotting results demonstrated that the Mincle mRNA and protein level were down-regulated in siRNA transfection group (siRNA-Mincle) and upregulated in plasmid transfection group (OE-Mincle) in BMDM cells (Fig. 6A-D). Therefore, we co-cultured the transfected BMDM cells with MES cells and found that compared with HG-treated co-culture cells, the mRNA and protein levels of inflammatory cytokines and activation of NF-κB in MES cells were inhibited and activated in siRNA-Mincle group and OE-Mincle group, respectively (Fig. 6E-L). Notably, overexpression of Mincle in BMDM reversed the inflmmatory response in co-cultured A&P-treated MES cells, suggesting that Mincle is the key to A&P to improve mesangial cell damage. All the data suggested that the anti-inflammatory role of A&P in MES cells is dependent on inhibition of Mincle/Card9/NFκB signaling pathway in BMDM.

## Discussion

Diabetic nephropathy is one of the most important causes of end-stage renal disease, and its incidence is increasing year by year, posing a serious threat to human health [26]. The progression of DN is associated with long-term hyperglycemia. Chronic hyperglycemia leads to disruption of hemodynamic and chronic metabolic pathways, both of which regulate different signaling pathways and cytokines that lead to kidney damage [27, 28]. The most characteristic pathological changes of DN are mainly glomerular hypertrophy, basement membrane thickening, glomerular mesangium and tubulointerstitial matrix expansion, and ultimately manifested as glomerulosclerosis and interstitial fibrosis [29, 30]. The pathogenesis of DN is complex, and to date, the progression of the disease has been clinically delayed by lowering the patient’s blood glucose levels. However, there is a lack of specific treatments for DN [5, 31]. Therefore, futher study on the pathogenesis of DN and development of targeted drugs is exceedingly important for DN treatment.

Nowadays, a large number of evidences demonstrate that inflammation plays an important role in the pathogenesis of DN [32]. Macrophages are the main inflammatory immune cells in kidney of DN, them can recruit other immune cells to mediate the renal inflammatory response. Affected by the microenvironment, macrophages differentiate into pro-inflammatory M1 macrophage and anti-inflammatory M2 macrophage. M1 macrophages and M2 macrophages are in a balance of pro- and anti-inflammatory in the immune inflammatory response. In many chronic inflammatory diseases, this balance is broken down, and M1 macrophages will continue to activate and aggravate the inflammatory response, releasing a large number of inflammatory factors such as IL-1β, TNF-α, and IL-6. Previous studies in human specimens and animal models of DN have found a significant increase in M1 macrophages and a significant decrease in M2 macrophages in DN [33, 34]. Briefly, M1/M2 ratio imbalance is a major cause of DN, which may be a new target for the treatment of DN.

M1 macrophages are triggered by the pattern recognition receptor Mincle, which contributes to the progression of acute kidney injury. Mincle is expressed primarily in macrophages, but also in dendritic cells, neutrophils, and T cells. Lin L. Lv et al. confirmed that the pathogenic role of Mincle in AKI: in the early stage of AKI, Mincle expression was significantly induced in infiltrating macrophages through the TLR4/NFκB signaling pathway [19, 35]. Studies have reported that Mincle/Syk/Card9 signal axis is critical in the development and progression of ocular autoimmune diseases [36]. In addtion, other studies have confirmed that Mincle can also activate NF-κB family transcription factors via Syk- and card9-dependent pathways [37]. Activated NF-κB translocation undergoes nuclear translocation and regulates the transcription of various factors, which in turn produce various chemokines and inflammatory factors. Although the pathogenic mechanism of Mincle have been studied in experimental models of AKI and UUO, they have not been reported in DN. Intriguingly, in our study, high expression of Mincle in the kidney of DN mice was observed by Real-time PCR and Western botting. Moreover, Mincle was expressed mainly in infiltrating macrophages in the kidney of DN mice, suggesting that Mincle is important for pathogenicity in DN. Therefore, reducing the expression of Mincle may inhibit the inflammatory response mediated by macrophages. Unfortunately, there are no effective Mincle inhibitors available to downregulate Mincle. Therefore, we intend to investigate whether A&P can improve the renal inflammation of DN through Mincle.

A&P has been used extensively in previous studies for the treatment of chronic kidney disease. We previously found that A&P can effectively improve colonic mechanical barrier dysfunction and inflammation, and improve impaired renal function. Importantly, we found in our preliminary studies that A&P alleviated cisplatin-induced AKI via inhibiting the Mincle maintained macrophage inflammation [24]. As a result, a signifificant decrease in the expression of Mincle in DN kidneys could be examined after A&P administration. These results have also demonstrated in in vitro experiments that A&P effectively down-regulates the expression of Mincle in high glucose-stimulated macrophages. We then examined the activation of NF-κB signaling, as well as the expression and secretion of many inflammatory factors such as TNF- α, IL-1β and IL-6. All the results showed that A&P can reduce the activation of NF-κB, and the expression and secretion of inflammatory cytokines in DN kidney or HG-induced BMDM cells. In the meantime, we observed that A&P treatment could improve renal function and relieve renal injury in DN mice.

In our study, this finding prompted us to explore the inhibition of Mincle-associated inflammatory responses by A&P treatment of target cells as a feasible strategy to delay DN. Macrophages are the major effector cells in various inflammatory reactions, as well as renal mesangial cells are also important components of the renal inflammation process involved in DN. Therefore, in this study, we established a co-culture model of BMDM cells and MES cells in order to mimic the state of cells in vivo. We found that the protein levels of p-NFκB, TNF-α, IL-1β and IL-6 were markedly higher in co-culture group than in the MES group, suggesting that macrophages can further intensify the inflammation response in mesangial cells under high glucose stimulation. So as to further study the mechanism of high glucose-induced macrophage activation and pro-inflammatory response, we blocked and overexpressed mincle by siRNA and plasmid transfection in the A&P-treated HG-induced BMDM cell group. Additionally, this further confirms that knockdown of Mincle in macrophages in vitro reduces the inflammatory response in HG-induced MES. Conversely, overexpression of Mincle in macrophages can upregulate the inflammatory response in HG-induced MES, and reverse the inflmmatory responses in co-cultured A&P-treated MES cells, suggesting a key role for Mincle in ameliorating mesenchymal cell injury by A&P. We believe that the anti-injury role of A&P on MES cells is dependent on the inhibition of Mincle pathway in macrophage.

## Conclusions

**Fig. 7:**
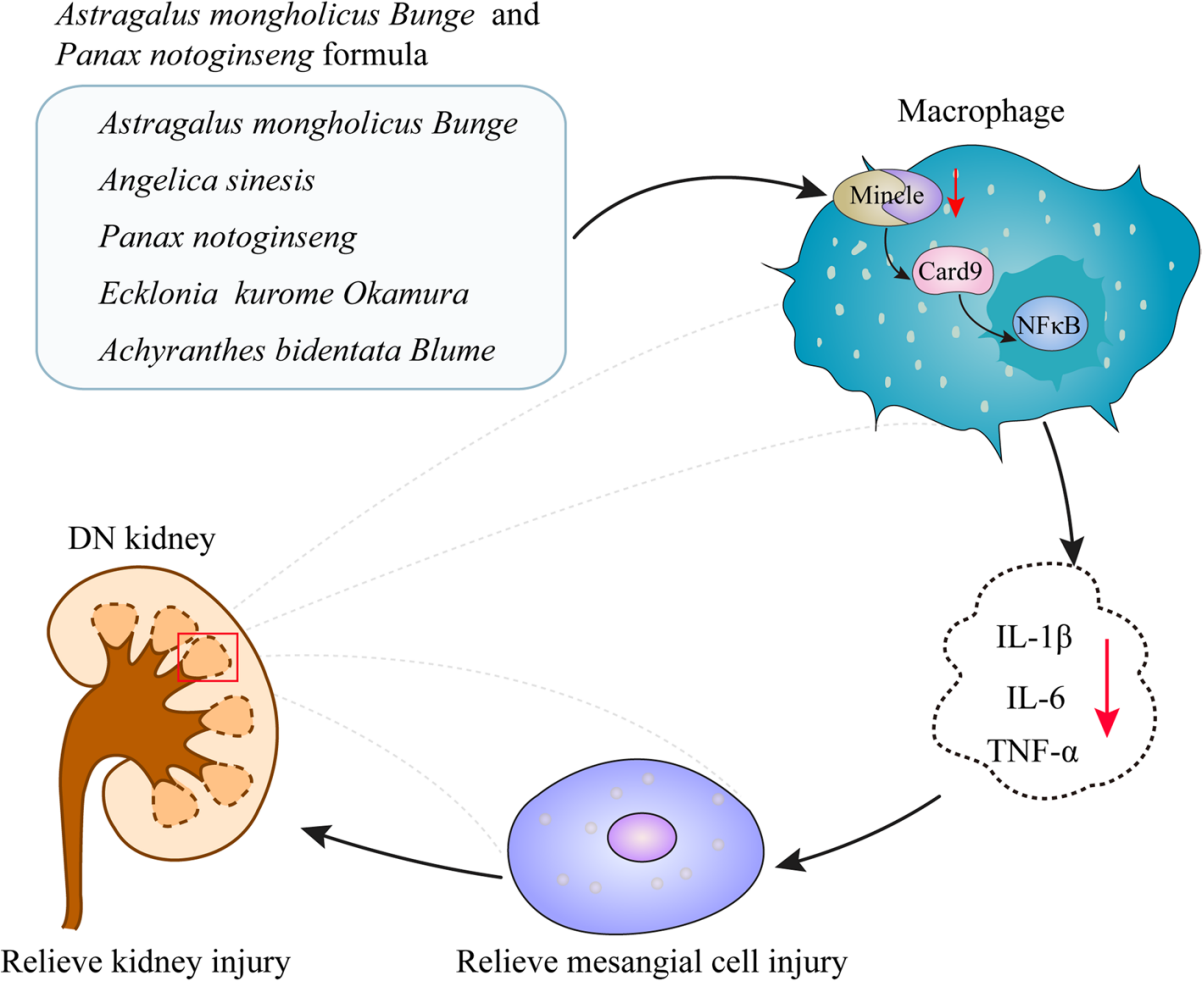
A&P relieved mesangial injury in kidney of diabetic nephropathy through inhibiting Mincle/Card9/NFκB signaling pathway in macrophage

In summary, our study found that A&P is effective in reducing renal pathological damage and improving renal function and inflammation in diabetic nephropathy by a mechanism mainly related to the inhibition of the Mincle/Card9/NFκB signaling pathway (Fig. 7). In short, A&P inhibits the activation of downstream Card9 and NF-κB by down-regulating the expression of Mincle in macrophages, thus reducing the secretion of inflammatory factors. As a result, inflammatory factors stimulated fewer renal interstitial cells, thereby alleviating interstitial cell damage and further reducing renal injury in diabetic nephropathy. Finally, these findings provide a new theoretical basis for the treatment of diabetic nephropathy with A&P.

## Supplementary Information

**Additional file 1: Supplementary Fig. 1** the expression of Mincle in BMDM cells after transfection of si-RNA and plasmid DNA. a-b, the protein level of Mincle in BMDM cells detected by western boltting analysis. (The data are expressed as the mean ± SD. Compared with NC group, *P < 0.01. Compared with HG group, ^#^*P* < 0.01. Compared with NC group, ^##^*P* < 0.05)

**Additional file 2.**

## Data Availability

The datasets used and analyzed during the current study are available from the corresponding author on reasonable request.

## Abbreviations

DN: Diabetic Nephropathy
A&P: *Astragalus mongholicus* Bunge and Panax notoginseng Formula
BMDM: Bone Marrow-Derived Macrophages cells
MES: Mouse Mesangial cells
RAAS: Renin–angiotensin–aldosterone system
CKD: Chronic Kidney Disease
ESRD: End-stage Renal Disease
AKI: Acute Kidney Injuery
PVDF: Polyvinylidene Fluoride
RPMI: Roswell Park Memorial Institute
OCT: Optimal Cutting Temperature compound
ELISA: Enzyme Linked Immunosorbent Assay
PBS: Phosphate Buffered Saline
