# Supplementary figures and images for: Astragalus mongholicus Bunge and Panax notoginseng formula (A&P) improves renal mesangial cell damage in diabetic nephropathy by inhibiting the inflammatory response of infiltrated macrophages
Source: BMC Complement Med Ther. 2022 Jan 20;22:17. doi: 10.1186/s12906-021-03477-x (PMC8781170; doi:10.1186/s12906-021-03477-x)

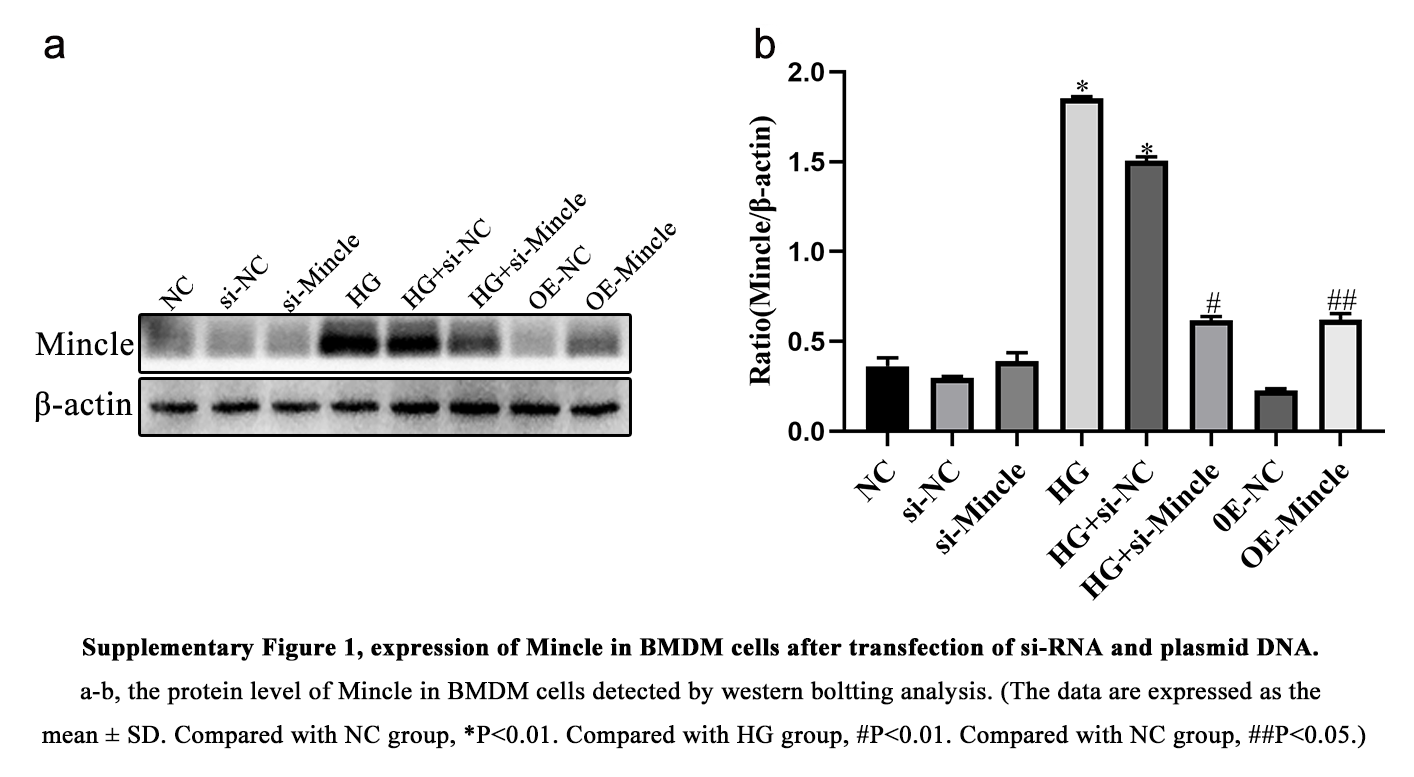

Supplement: Supplementary file 1 — Additional file 1: Supplementary Fig. 1 the expression of Mincle in BMDM cells after transfection of si-RNA and plasmid DNA. a-b, the protein level of Mincle in BMDM cells detected by western boltting analysis. (The data are expressed as the mean ± SD. Compared with NC group, *P < 0.01. Compared with HG group, #P < 0.01. Compared with NC group, ##P < 0.05) [file 12906_2021_3477_MOESM1_ESM.tif]
