# Supplementary material for: Astragalus mongholicus Bunge and Panax notoginseng formula (A&P) improves renal mesangial cell damage in diabetic nephropathy by inhibiting the inflammatory response of infiltrated macrophages
Source: BMC Complement Med Ther. 2022 Jan 20;22:17. doi: 10.1186/s12906-021-03477-x (PMC8781170; doi:10.1186/s12906-021-03477-x)
Supplement: Supplementary file 2 — Additional file 2. [file 12906_2021_3477_MOESM2_ESM.pdf]

The RAW data of WB results

Figure 2

NFκB

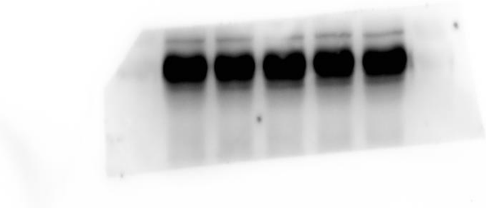

p-NFκB

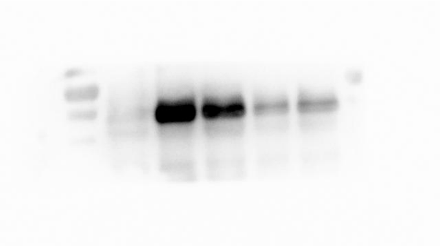

IL-6

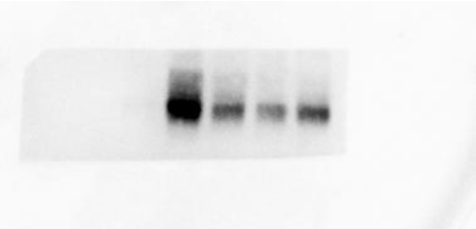

IL-1β

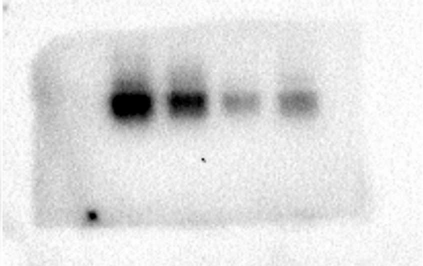

TNF-α

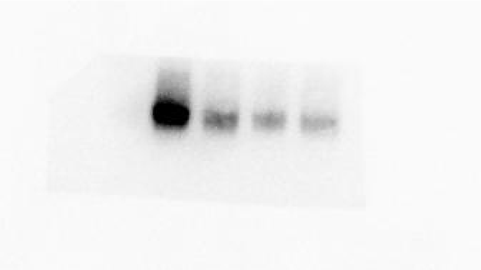

β-actin

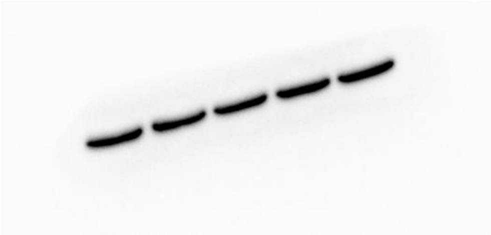

The above images are the RAW data of the WB results in Figure 2, each image corresponds to the WB band in Figure 2.

Figure 3

β-actin

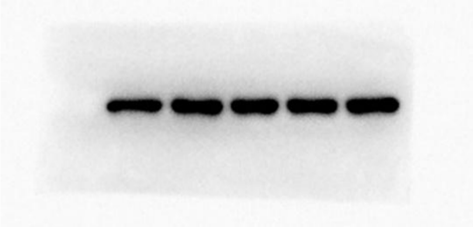

Mincle

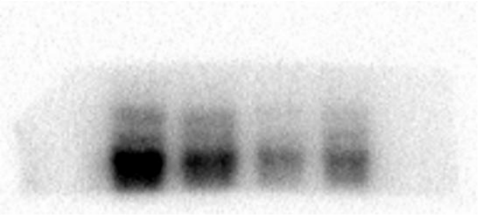

The above images are the RAW data of the WB results in Figure 3, each image corresponds to the WB band in Figure 3.

Figure 4

p-NFκB

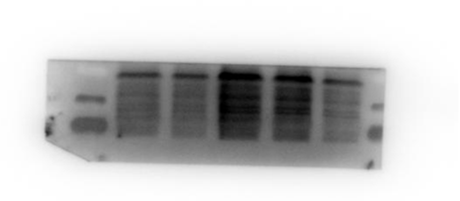

NFκB

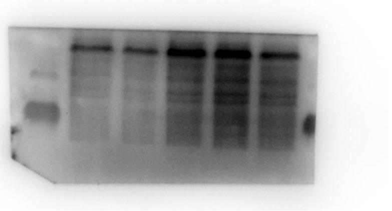

Card9

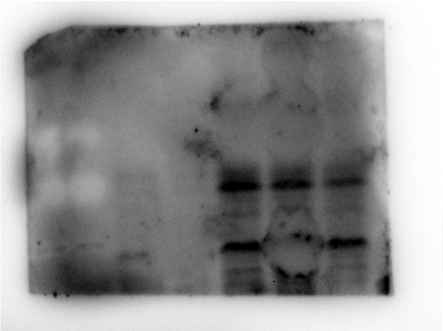

Mincle

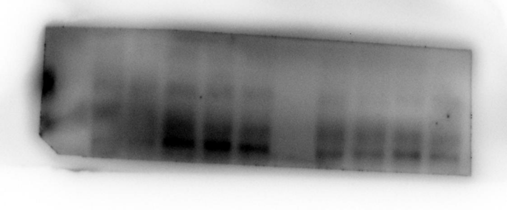

TNF-α

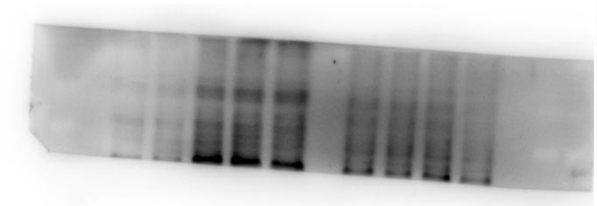

IL-1β

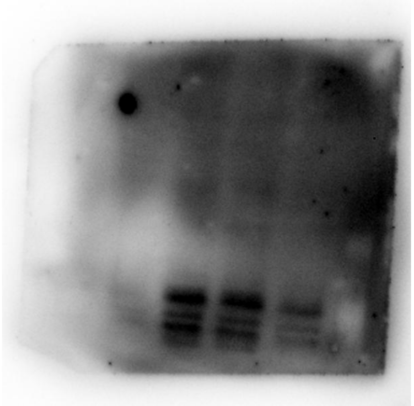

β-actin

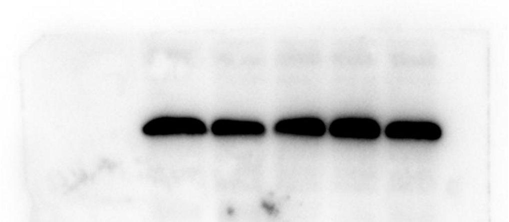

The above images are the RAW data of the WB results in Figure 4, each image corresponds to the WB band in Figure 4.

Figure 5

p-NFκB

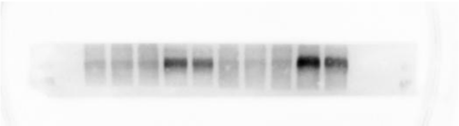

NFκB

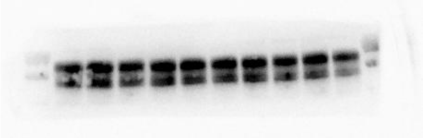

IL-1β

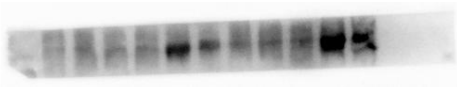

IL-6

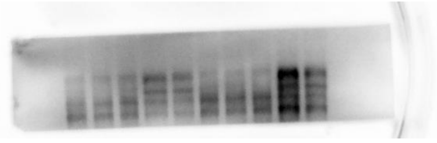

TNF-α

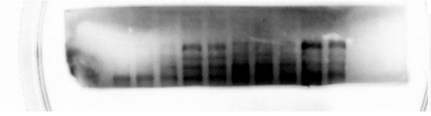

β-actin

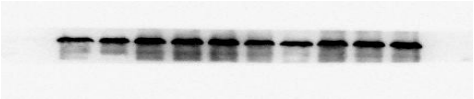

The above images are the RAW data of the WB results in Figure 5, each image corresponds to the WB band in Figure 5.

Figure 6

p-NFκB

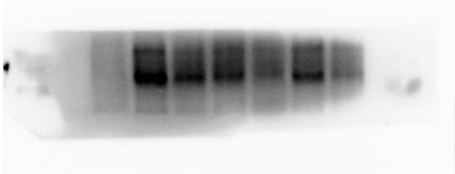

NFκB

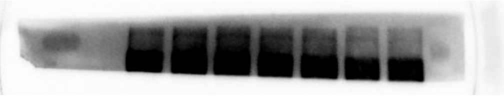

Card9

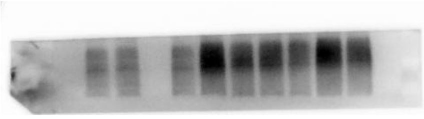

Mincle

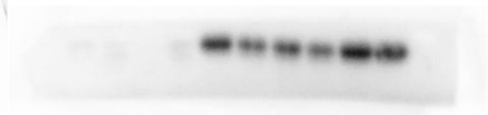

IL-1β

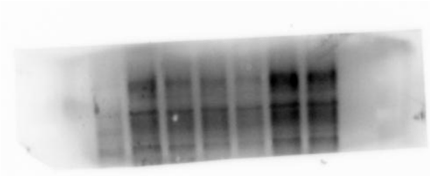

IL-6

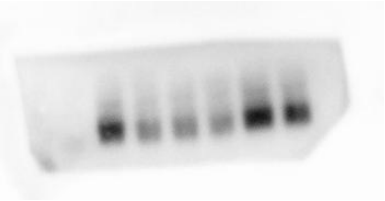

TNF-α

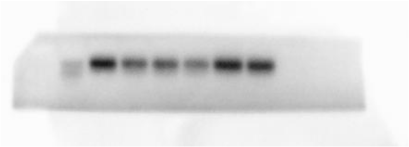

β-actin

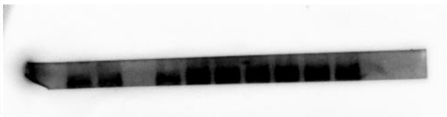

β-actin

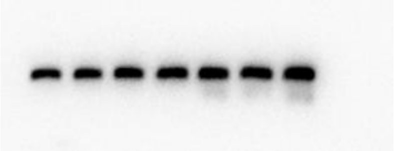

The above images are the RAW data of the WB results in Figure 6, each image corresponds to the WB band in Figure 6.

Supplementary figure

Mincle

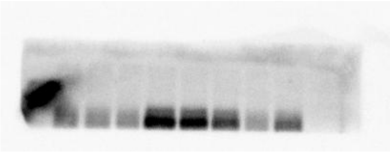

β-actin

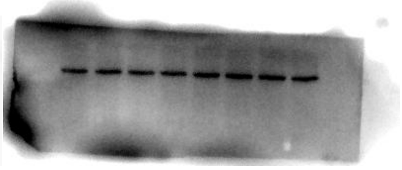

The above images are the RAW data of the WB results in Supplementary figure1, each image corresponds to the WB band in Supplementary figure1.
